# Correlation between Preoperative MRI Parameters and Oswestry Disability Index in Patients with Lumbar Spinal Stenosis: A Retrospective Study

**DOI:** 10.3390/medicina59112000

**Published:** 2023-11-14

**Authors:** Radu Caprariu, Manuel D. Oprea, Dan V. Poenaru, Diana Andrei

**Affiliations:** 1 Department of Radiology and Medical Imaging, University of Medicine and Pharmacy “Victor Babes”, 300041 Timisoara, Romania; radu.caprariu@umft.ro; 2 Department of Traumatology and Orthopedics, University of Medicine and Pharmacy “Victor Babes”, 300041 Timisoara, Romania; danvpoenaru@gmail.com; 3 Department of Balneology, Medical Rehabilitation and Rheumatology, University of Medicine and Pharmacy “Victor Babes”, 300041 Timisoara, Romania; andreidiana81@gmail.com

**Keywords:** degenerative disease, MRI, lumbar stenosis

## Abstract

*Background and Objectives*: Lumbar spinal stenosis (LSS) is a degenerative condition posing significant challenges in clinical management. Despite the use of radiological parameters and patient-reported outcome measures like the Oswestry Disability Index (ODI) for evaluation, there is limited understanding of their interrelationship. This study aimed to investigate the correlation between preoperative MRI parameters and ODI scores in patients with LSS undergoing surgical treatment. *Materials and Methods*: A retrospective analysis was conducted on 86 patients diagnosed with LSS over a 5-year period. Preoperative MRI measurements, including the cross-sectional area of the psoas muscle, lumbar canal stenosis, neural foramina area, and facet joint osteoarthritis, were assessed. ODI scores were collected preoperatively and at a 1-year follow-up. Statistical analyses were performed using IBM SPSS Statistics software (version 26). *Results*: Weak to moderate correlations were observed between certain MRI parameters and ODI scores. The initial ODI score had a weak positive correlation with the severity of lumbar canal stenosis according to Schizas criteria (rho = 0.327, *p* = 0.010) and a moderate negative correlation with the relative cross-sectional area of the psoas muscle (rho = −0.498, *p* = 0.000). At 1-year follow-up, the ODI had a weak negative correlation with the relative cross-sectional area of the psoas muscle (rho = −0.284, *p* = 0.026). *Conclusions*: While the severity of LSS showed a weak correlation with initial ODI, it was not a predictor of 1-year postoperative ODI. Furthermore, although the cross-sectional area of the thecal sac, the sagittal area of the neural foramen, and the grade of facet joint osteoarthritis influence the imagistic severity, none of them correlate with ODI. These findings underscore the need for a comprehensive model that integrates multiple imaging and clinical parameters for a holistic understanding of LSS and its functional outcomes.

## 1. Introduction

Lumbar spinal stenosis (LSS) is a degenerative spinal condition that poses increasing challenges for clinicians due to its multifactorial etiology and the complexity of its clinical presentation. Characterized by a narrowing of the spinal canal, LSS often leads to neurogenic claudication, radicular symptoms, and significant functional impairment. With an aging global population, the prevalence of LSS is expected to rise, making it an increasingly critical public health concern [1,2,3]. Surgical intervention, such as decompressive laminectomy or spinal fusion, is frequently recommended for moderate to severe cases unresponsive to conservative management [4].

Functional outcomes following surgery are traditionally evaluated using patient-reported outcome measures (PROMs) like the Oswestry Disability Index (ODI). The ODI is a validated, reliable tool for assessing disability related to low back pain and has been extensively used in the context of LSS [5,6]. However, there is an increasing recognition of the need to integrate objective radiological parameters with these subjective outcome measures to provide a more comprehensive understanding of postoperative recovery and functional status [7,8].

The psoas muscle, a major muscle of the trunk, has been implicated in spine mechanics and low back pain [9,10]. Recent advances in magnetic resonance imaging (MRI) have enabled the precise quantification of the cross-sectional area of the psoas muscle. Previous studies have suggested that the size and quality of the psoas muscle may be associated with functional outcomes in various spinal disorders [11,12]. However, limited research has been conducted to explore the correlation between the cross-sectional area of the psoas muscle as measured by MRI and functional outcomes, specifically the ODI score, in patients with LSS undergoing surgical intervention.

This retrospective study aims to investigate the relationship between preoperative MRI measurements (the cross-sectional area of the psoas muscle, central stenosis, and facet joint degeneration) and the ODI values reported preoperatively and postoperatively (1-year follow-up). By examining these correlations, this study seeks to contribute valuable insights into the prognostic factors affecting functional recovery following surgical intervention for LSS.

## 2. Materials and Methods

A retrospective study was conducted on a lot of 86 patients diagnosed with LSS in a clinical county hospital over a period of 5 years (January 2018 to January 2023). Institutional approval was obtained from the Scientific Research Committee (No. 60/22.12.2021). At the initial evaluation and surgical treatment, 122 participants were enrolled and their data were processed. At the 1-year follow-up, a number of 36 cases were invalidated due to either missing follow-up data or other medical conditions that would have biased our data. A final 86 participants (37 men and 49 women) with a mean age of 54.8 years (ranging from 32 to 82 years, std. dev. 14.40) were further investigated.

Upon admission, all patients were subjected to a preoperative MRI investigation of the lumbar spine. MRI measurements were performed and noted as follows: the cross-sectional area of the psoas muscle, the area of the highest degree of lumbar canal stenosis (on axial T2 sequence), the area of the neural foramina (on sagittal T2 sequence), and facet joint degeneration (*n* = 516).

MRI images were independently analyzed by two of the authors, a radiologist and a spinal surgeon, using DICOM Horos software (version 3.3.6 for OsX, Apple, Cupertino, CA). In this study, the relative cross-sectional area of the psoas muscle (rCSA) was used as previously described in another study authored by the investigators [13]. These values represent a ratio between the mean cross-sectional area of the psoas muscle and the cross-sectional area of the L4 lower end-plate [13]. The area of the neural foramen was measured bilaterally (L3-L4 to L5-S1) by manually outlying the fat and neural structure. The smallest value was chosen for further investigation. Facet joint osteoarthritis was evaluated bilaterally from L3-L4 to L5-S1 according to the Weishaupt et al. description in a 4-grade manner [14], and the highest grade observed was noted. All the values manually measured by the two investigators were summed, and their mean was used for further investigation.

The number of levels involved in the stenotic process (i.e., from 1 to 5) was also noted. The severity of the canal stenosis was scored on axial MRI images according to Schizas criteria: A—no/minor; B—moderate; C—severe; D—extreme [15]. The intra- and inter-observer coefficients were 0.80 for rCSA measurement, 0,95 for lumbar lordosis, 0.72 for facet joint osteoarthritis, 0.83 for the area of neural foramen, and 0.77 for the severity of stenosis. The coefficients were confirmed by kappa analysis to be sufficient.

After diagnosis and other specific steps, all patients underwent surgical treatment mainly by decompressive laminectomy and spinal fusion (TLIF) and in several cases by hemilanotomy/foraminotomy. Surgery was performed by the same surgical team in the same surgical center and using identical implant types. All participants were referred by a previous ambulatory examination, and the surgical indication was based on the presence of radiculopathy with neurogenic claudication, associated pain, or severe disability. The cases with central stenosis or bilateral lateral stenosis were treated by decompression and fusion. Whenever signs of instability or abnormal sagittal balance were present, decompression and TLIF were chosen. Only a few selected cases were treated by hemilaminectomy/foraminotomy alone (when the compression could be clearly objectified and no signs of instability were present).

Patients were evaluated preoperatively and postoperatively (1-year follow-up) via the ODI questionnaire. The ODI is a widely used, self-administered questionnaire that quantifies a patient’s perceived disability level in various aspects of daily living. It offers a multidimensional approach to assessing pain, function, and health status, thus providing a comprehensive view of a patient’s disability level. Preoperative functional status was assessed using the ODI questionnaire by the supervising physician together with the patient. Questions about the intensity of pain, ability to lift, ability to care for oneself, ability to walk, ability to sit, ability to stand, social life, sleep quality, and ability to travel were prompted.

For each question, there is a possible 5 points; 0 for the first answer, 1 for the second answer, etc. The total points for the 10 questions are added together and rated as follows: 0–4, no disability; 5–14, mild disability; 15–24, moderate disability; 25–34, severe disability; 35–50, completely disabled [16].

All data were analyzed using IBM SPSS Statistics software (IBM SPSS Statistics for OsX, Version 26.0, IBM Corp., Armonk, New York). Results were presented as means ± standard deviations. The normal distribution for the monitored parameters was checked using the chi-square and Shapiro–Wilk methods. The variables were analyzed using Pearson parametric and non-parametric Spearman correlations.

## 3. Results

### 3.1. Imaging Parameters

**Figure 1:**
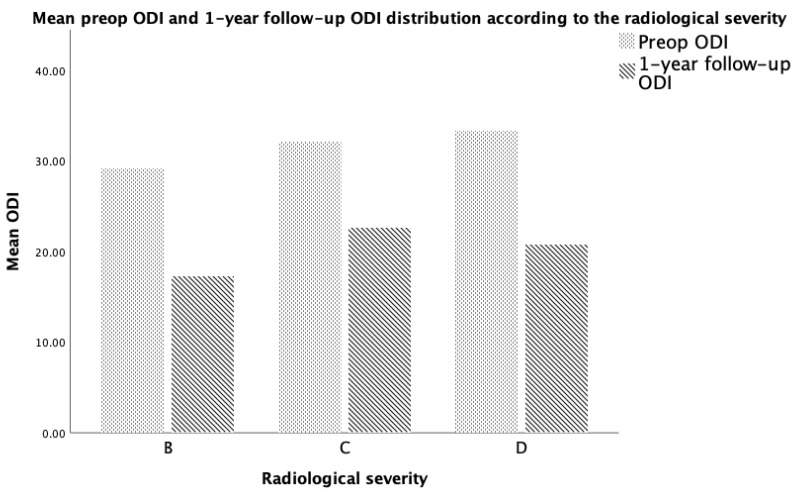
Distribution of PROM according to the imagistic severity of their LSS.

In total, 516 facet joints and neural foramina were assessed on MRI scans from 86 individuals. Grade III facet joint osteoarthritis (FJOA) was observed in 40 cases (46.5%) while grade II accounted for 38 cases (44.2%). In terms of radiologic severity of lumbar canal stenosis, 44 patients (accounting for 51.2%) had moderate stenosis, while mild and severe were observed in an equal number of patients (21 patients accounting for 24.4% each). The mean rCSA was 0.7734 (±0.185), ranging from 0.46 to 1.12, while the sagittal area of the neural foramen (NFA) ranged from 0.39 cm^2^ to 1.58 cm^2^ (mean 0.7863, ±0.273). The mean area of the thecal sac (TSA) as measured on the axial T2 images at the most narrowed part was 1.304 cm^2^ (ranging from 0.561 to 2.713, ±0.511). A summary of these parameters can be observed in Figure 1 and Table 1 and Table 2.

### 3.2. Patient-Reported Outcome Measure—ODI

According to the initial preoperative functional score as assessed by ODI, 59 individuals (68.6%) had a severe disability, 21 were completely disabled (24.4%), and 6 were moderately disabled (7.0%). No statistical difference in the disability index was noted between men and women.

At the 1-year follow-up, according to the ODI questionnaire, 47 individuals reported moderate disability (54.7%), 22 reported severe disability (25.6%), 1 reported complete disability (1.2%), and 1 reported no disability (1.2%). Table 3 summarizes the scores assessed through these PROMs.

### 3.3. ODI Functional Score Correlation with Imagistic Parameters

**Figure 2:**
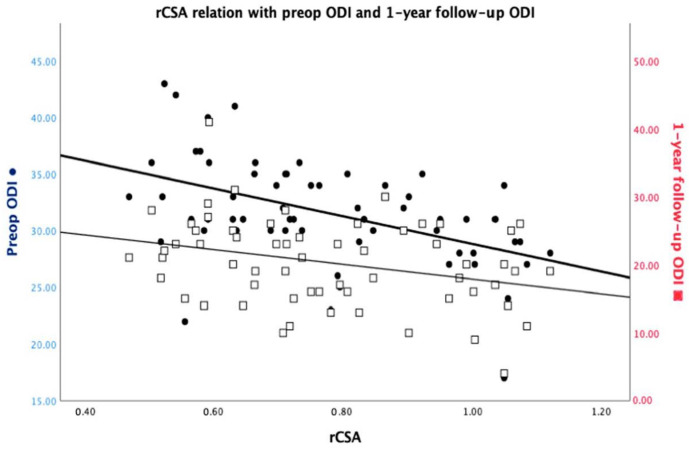
rCSA interrelationship with the preoperative ODI and the 1-year follow-up ODI.

The initial ODI score had a weak positive correlation with the imagistic severity of LSS as assessed according to Schizas criteria (rho = 0.327, *p* = 0.010) and a moderate positive correlation with the 1-year follow-up ODI score (rho = 0.495, *p* = 0.000). There was a moderate negative correlation between the initial ODI and the relative CSA of the psoas muscle (rho = −0.498, *p* = 0.000). When adjusting for age, the initial ODI was observed to exhibit a correlation only with the 1-year follow-up ODI (rho = 0.422, *p* = 0.001) and the relative CSA (rho = −0.375, *p* = 0.003) (Figure 2). There were no other statistically significant correlations (Table 4).

The 1-year follow-up ODI had a weak negative correlation with the relative CSA of the psoas muscle (rho = −0.284, *p* = 0.026). Besides the weak positive correlation with the initial ODI, the 1-year ODI had no other statistically significant correlations, even when adjusting for age (Table 4).

The imagistic severity of the canal narrowing had a moderate negative correlation with the area of the thecal sac (rho = −0.466, *p* = 0.000) that was also observed when adjusting for age (rho = −0.549, *p* = 0.000). The imagistic severity had a weak negative correlation with the sagittal area of the neural foramen (rho = −0.335, *p* = 0.008) that was also observed when adjusting for age (rho = −0.373, *p* = 0.008). The imagistic severity had a weak positive correlation with facet joint osteoarthritis (rho = 0.275, *p* = 0.032) and the initial ODI (rho = 0.327, *p* = 0.010), but no correlations were observed when adjusting for age.

There was a moderate negative correlation between the sagittal area of the neural foramen and the facet joint OA grade (rho = −0.439, *p* = 0.000) that was also observed when adjusting for age (rho = −0.306, *p* = 0.017). There were no other statistically significant correlations (Table 4).

## 4. Discussion

The need for a comprehensive understanding of LSS by corroborating objective imagistic parameters alongside clinical ones is imperative as the medical management of patients with LSS poses a great socio-economic burden [3]. Although modern imagistic modalities can detect early changes in the lumbar spine, it is still difficult to provide a specific diagnosis and subsequent treatment for a large proportion of patients as LSS has a myriad of clinical expressions and its outcome may be hard to predict. A possible explanation might be that nearly all lumbar structures can be a source of low back pain [17,18]. Another challenge comes from the frequent association with other pathologies, e.g., osteoarthritis in the elderly, which encumbers the differential diagnosis and slows the functional outcome.

The exploration of lumbar spine stenosis using imagistic parameters and patient-reported outcome measures (ODI) has been investigated by several studies, with mixed results [13,15,18,19,20,21]. The prevalence of grade III and II facet joint osteoarthritis (FJOA) reflects a significant burden of facet joint degeneration within the studied population. This aligns with the existing literature that posits FJOA as a common radiological finding in individuals with lumbar spinal disorders [22,23,24,25]. FJOA has a major implication in LSS as it impacts the narrowing of either the subarticular recess or the foramen through an osteophyte. A study conducted by Lee et al. concluded that there are limitations during a preoperative imagistic evaluation of patients considered for motion-sparing techniques in lumbar spinal surgery. Although osteoarthritic alterations can directly compromise the root, they pose a great difficulty in objectifying them through a 2D imagistic quantitative parameter.

In our analysis, we found a statistically significant moderate negative correlation between facet joint osteoarthritis (FJOA) and the sagittal area of the neural foramen, as indicated by a correlation coefficient of rho = −0.439. However, it is worth noting that this correlation did not extend to the initial Oswestry Disability Index (ODI) scores, suggesting that the severity of FJOA alone may not be a reliable predictor of initial functional disability in this study lot because the stenosis might have been only central. In a study conducted on 63 individuals by Sirvanci et al., the authors concluded that there was no correlation between ODI and imagistic appearances [19]. The previously mentioned study used mainly qualitative parameters, while a mix of quantitative and qualitative assessment tools were used in this study in an effort to eliminate any errors related to this type of data. In this study, although there was a positive correlation between the initial ODI and the radiologic severity of the LSS and FJOA assessed qualitatively, the rho correlation coefficient was low. Furthermore, the correlation did not extend to any quantitative parameters such as the cross-sectional area of the thecal sac or the sagittal area of the neural foramen which should increase the severity grading. Maataoui et al. did not find any correlation between bilateral FJOA and ODI (only one-sided correlation) in their study, but they statistically analyzed all the facet joints, while in this study, only the most degenerated facet joint value was further analyzed [18]. Another recent study conducted by Wu ZX et al. on 196 individuals found a correlation between the imagistic parameters and functional scores [26]. Furthermore, they concluded that patients with severe root compression have more severe clinical symptoms than the rest.

The patient-reported outcomes, as assessed by the ODI, underscore the functional disability associated with these anatomical alterations. The shift in ODI scores from preoperative to 1-year follow-up indicates a varied degree of functional recovery, which is a critical aspect of patient-centered care in spinal disorders. Although there were several cases with no improvement in the ODI score, these reflect the actual difficulty in surgically treating LSS as cases of failed back surgery are frequently reported [27,28,29,30].

The correlations between ODI scores and imagistic parameters like the relative CSA (initial: rho= −0.498, *p* = 0.000/1-year: rho = −0.284, *p* = 0.026) of the psoas muscle echo the intricate interplay between anatomical and functional dimensions. An even more interesting aspect observed in this study is that age does not appear to influence the relationship between rCSA and initial ODI. As previously investigated by other authors, the morphological status of the psoas muscle can be an indicator of good functional rehabilitation after spinal surgery [31,32].

This study contributes to the growing literature on the confluence of imaging parameters and functional outcomes in lumbar spine pathologies by studying both quantitative and qualitative imagistic parameters in relation to PROMs before and after surgical treatment. There are conflicting views in the literature regarding minimal decompression alone through hemilaminotomy/foraminotomy vs. a more extensive decompression in terms of long-term outcome [33,34,35]. Such a comparison was not one of this study’s objectives but might have influenced its results.

The imagistic severity grading system as proposed by Schizas is based on the morphological appearance of the dural sac in axial T2-weighted MRI images of the lumbar spine [15]. It analyzes the arrangement of nerve roots in relation to the cerebrospinal fluid and thus has the advantages of not requiring specific measurement tools and being easily applicable to daily practice. As observed in this study, age does not influence the relationship between the severity classes and the morphological changes in the structures that are part of the classification criteria. Both the cross-sectional diameter of the thecal sac and the sagittal diameter of the neural foramen were correlated with the imagistic severity after adjusting for age. But although it lacks clinical correlation, as observed in this study after controlling for age, this classification tool provides a valuable homogeneous common language between physicians [36].

The limitation of this study is the relatively small number of patients, although relatively equal [37] or smaller [38] numbers of patients were included in previous prospective studies. Future studies employing larger cohorts are warranted to validate these findings and to further investigate the complex interplay between anatomical, clinical, and functional dimensions in lumbar spine pathologies.

This study showed that the ODI score is in a weak correlation with the imagistic severity of the LSS but the severity is not a predictor of 1-year functional outcome as assessed through ODI. Furthermore, although the imagistic severity includes the area of the thecal sac, this parameter alone does not correlate with ODI; the same is the case with the sagittal area of the neural foramen. This implies that when trying to assess a patient with such complex modifications as found in LSS, one needs to integrate multiple imagistic parameters in order to better understand the severity. The correlations revealed may provide a foundation for further exploration into the underlying mechanisms associating anatomical changes with functional disabilities. However, the study’s limitations, including the modest sample size, imply the need for larger cohort studies to validate these findings and to unravel the complex interplay between the anatomical, clinical, and functional dimensions of lumbar spine pathologies.

## 5. Conclusions

The results of our study indicate that certain imagistic markers like the severity of lumbar spinal stenosis and the relative cross-sectional area of the psoas muscle exhibit weak to moderate correlations with both preoperative and 1-year follow-up ODI scores. The study found that while the severity of canal stenosis as measured by Schizas criteria weakly correlated with initial ODI, it was not a predictor of the 1-year postoperative ODI. This underlines the inherent limitations of relying solely on radiological severity for predicting functional outcomes in LSS.

Other imaging parameters like the sagittal area of the neural foramen and the facet joint osteoarthritis grade were found to have no statistically significant correlations with the ODI.

## Figures and Tables

**Table 1:** Summary of the imagistic parameters assessed.

Facet Joint OA		Lumbar Canal Stenosis	
Weishaupt Grading	No. Cases (Frequency)	Schizas Grading	No. Cases (Frequency)
0	0	A	0
1	8 (9.3%)	B	21 (24.4%)
2	38 (44.2%)	C	44 (51.2%)
3	40 (46.5%)	D	21 (24.4%)

A—normal; B—moderate; C—severe; D—extreme.

**Table 2:** Summary of the imaging parameters assessed.

Imaging Parameters—Descriptive Statistics				
	Min	Max	Mean	Std. Dev.
Age	32	82	54.80	14.400
rCSA	0.46	1.12	0.7734	0.18506
TSA	0.561	2.713	1.304	0.511
NFA	0.39	1.58	0.7863	0.27302

rCSA—relative cross-sectional area of the psoas muscle; TSA—thecal sac area; NFA—neural foramen area.

**Table 3:** Initial and 1-year follow-up ODI mean score distribution.

ODI Mean Score Distribution in Disability Levels								
	Initial ODI Disability Level			1-Year Follow-Up ODI Disability Level				
	Moderate	Severe	Completely	No disability	Mild	Moderate	Severe	Completely
	6 (7%)	21 (24.4%)	59 (68.6%)	1 (1.2%)	14 (16.3%)	47 (54.7%)	23 (26.7%)	1 (1.2%)
Mean ODI score	21.50	30.48	37.27	4	4.00	11.90	19.48	41.00

**Table 4:** Spearman correlations between imaging parameters and ODI score.

		Severity	Foramen	rCSA	Initial ODI	1-year ODI
Imagistic severity	*Rho*	1.000	−0.335 **	−0.012	0.327 *	0.180
NFA	*Rho*	−0.335 **	1.000	0.087	−0.057	−0.011
rCSA	*Rho*	−0.012	0.087	1.000	−0.498 **	−0.284 *
Initial ODI score	*Rho*	0.327 *	−0.057	−0.498 **	1.000	0.495 **
1-year follow-up ODI score	*Rho*	0.180	−0.011	−0.284 *	0.495 **	1.000

* *p* < 0.05, ** *p* < 0.01; NFA—neural foramen area; rCSA—relative cross-sectional area of the psoas muscle.

## Data Availability

Data are available on request due to restrictions (e.g., privacy or ethical restrictions).

## References

[1] Kalichman L., Cole R., Kim D.H., Li L., Suri P., Guermazi A., Hunter D.J. (2009). Spinal stenosis prevalence and association with symptoms: The Framingham Study. Spine J..

[2] Deyo R.A., Mirza S.K., Martin B.I., Kreuter W., Goodman D.C., Jarvik J.G. (2010). Trends, major medical complications, and charges associated with surgery for lumbar spinal stenosis in older adults. JAMA.

[3] Maeda T., Hashizume H., Yoshimura N., Oka H., Ishimoto Y., Nagata K., Takami M., Tsutsui S., Iwasaki H., Minamide A. (2018). Factors associated with lumbar spinal stenosis in a large-scale, population-based cohort: The Wakayama Spine Study. PLoS ONE.

[4] Weinstein J.N., Tosteson T.D., Lurie J.D., Tosteson A.N.A., Blood E., Hanscom B., Herkowitz H., Cammisa F., Albert T., Boden S.D. (2008). Surgical versus nonsurgical therapy for lumbar spinal stenosis. N. Engl. J. Med..

[5] Fairbank J.C., Pynsent P.B. (2000). The Oswestry Disability Index. Spine.

[6] Davidson M., Keating J.L. (2002). A comparison of five low back disability questionnaires: Reliability and responsiveness. Phys. Ther..

[7] Lee J.H., Lee S.H. (2014). Comparative analysis of clinical outcomes in patients with osteoporotic vertebral compression fractures (OVCFs): Conservative treatment versus balloon kyphoplasty. Spine J..

[8] Teichtahl A.J., Urquhart D.M., Wang Y., Wluka A.E., Wijethilake P., O’Sullivan R., Cicuttini F.M. (2015). Fat infiltration of paraspinal muscles is associated with low back pain, disability, and structural abnormalities in community-based adults. Spine J..

[9] Hodges P., Holm A.K., Hansson T., Holm S. (2006). Rapid atrophy of the lumbar multifidus follows experimental disc or nerve root injury. Spine.

[10] Zhang Y., Mandelli F., Mündermann A., Nüesch C., Kovacs B., Schären S., Netzer C. (2021). Association between fatty infiltration of paraspinal muscle, sagittal spinopelvic alignment and stenosis grade in patients with degenerative lumbar spinal stenosis. N. Am. Spine Soc. J..

[11] Tanimoto Y., Watanabe M., Sun W., Sugiura Y., Hayashida I., Kusabiraki T., Tamaki J. (2014). Sarcopenia and falls in community-dwelling elderly subjects in Japan: Defining sarcopenia according to criteria of the European Working Group on Sarcopenia in Older People. Arch. Gerontol. Geriatr..

[12] Wagner S.C., Sebastian A.S., McKenzie J.C., Butler J.S., Kaye I.D., Morrissey P.B., Vaccaro A.R., Kepler C.K. (2018). Severe Lumbar Disability Is Associated with Decreased Psoas Cross-Sectional Area in Degenerative Spondylolisthesis. Glob. Spine J..

[13] Caprariu R., Oprea M., Popa I., Andrei D., Birsasteanu F., Poenaru V.D. (2023). Cohort study on the relationship between morphologic parameters of paravertebral muscles, BMI and lumbar lordosis on the severity of lumbar stenosis. Eur. J. Orthop. Surg. Traumatol..

[14] Weishaupt D., Zanetti M., Boos N., Hodler J. (1999). MR imaging and CT in osteoarthritis of the lumbar facet joints. Skelet. Radiol..

[15] Lohman C.M., Tallroth K., Kettunen J.A., Lindgren K.A. (2006). Comparison of radiologic signs and clinical symptoms of spinal stenosis. Spine.

[16] https://www.aaos.org/globalassets/quality-and-practice-resources/patient-reported-outcome-measures/spine/oswestry-2.pdf.

[17] Zotti M.G.T., Boas F.V., Clifton T., Piche M., Yoon W.W., Freeman B.J.C. (2017). Does pre-operative magnetic resonance imaging of the lumbar multifidus muscle predict clinical outcomes following lumbar spinal decompression for symptomatic spinal stenosis?. Eur. Spine J..

[18] Maataoui A., Vogl T.J., Middendorp M., Kafchitsas K., Khan M.F. (2014). Association between facet joint osteoarthritis and the Oswestry Disability Index. World J. Radiol..

[19] Sirvanci M., Bhatia M., Ganiyusufoglu K.A., Duran C., Tezer M., Ozturk C., Aydogan M., Hamzaoglu A. (2008). Degenerative lumbar spinal stenosis: Correlation with Oswestry Disability Index and MR imaging. Eur. Spine J..

[20] Carragee E.J., Alamin T.F., Miller J.L., Carragee J.M. (2005). Discographic, MRI and psychosocial determinants of low back pain disability and remission: A prospective study in subjects with benign persistent back pain. Spine J..

[21] Herno A., Airaksinen O., Saari T. (1994). Computed tomography after laminectomy for lumbar spinal stenosis. Patients’ pain patterns, walking capacity, and subjective disability had no correlation with computed tomography findings. Spine.

[22] Yoshiiwa T., Miyazaki M., Notani N., Ishihara T., Kawano M., Tsumura H. (2016). Analysis of the Relationship between Ligamentum Flavum Thickening and Lumbar Segmental Instability, Disc Degeneration, and Facet Joint Osteoarthritis in Lumbar Spinal Stenosis. Asian Spine J..

[23] Battié M.C., Videman T., Gibbons L.E., Fisher L.D., Manninen H., Gill K. (1995). 1995 Volvo Award in clinical sciences. Determinants of lumbar disc degeneration. A study relating lifetime exposures and magnetic resonance imaging findings in identical twins. Spine.

[24] Wang A., Wang T., Zang L., Yuan S., Fan N., Du P., Wu Q. (2022). Quantitative Radiological Characteristics of the Facet Joints in Patients with Lumbar Foraminal Stenosis. J. Pain. Res..

[25] Lee J.C., Cha J.G., Yoo J.H., Kim H.K., Kim H.J., Shin B.J. (2012). Radiographic grading of facet degeneration, is it reliable?—A comparison of MR or CT grading with histologic grading in lumbar fusion candidates. Spine J..

[26] Wu Z.X., Xiao L., Zhao Q.L., Liu C., Sun H.Z., Geng Y., Jiang Y.J. (2023). Clinical significance and risk factors of redundant nerve root in patients with lumbar spinal stenosis. Zhongguo Gu Shang.

[27] Omidi-Kashani F., Hasankhani E.G., Ashjazadeh A. (2014). Lumbar spinal stenosis: Who should be fused? An updated review. Asian Spine J..

[28] Kovacs F.M., Urrútia G., Alarcón J.D. (2011). Surgery versus conservative treatment for symptomatic lumbar spinal stenosis: A systematic review of randomized controlled trials. Spine.

[29] Aghayev E., Mannion A.F., Fekete T.F., Janssen S., Goodwin K., Zwahlen M., Berlemann U., Lorenz T., Spine Tango Registry Group (2020). Risk Factors for Negative Global Treatment Outcomes in Lumbar Spinal Stenosis Surgery: A Mixed Effects Model Analysis of Data from an International Spine Registry. World Neurosurg..

[30] Oprea M., Popa I., Cimpean A.M., Raica M., Poenaru D.V. (2015). Microscopic assessment of degenerated intervertebral disc: Clinical implications and possible therapeutic challenge. In Vivo.

[31] Verla T., Adogwa O., Elsamadicy A., Moreno J.R., Farber H., Cheng J., Bagley C.A. (2016). Effects of Psoas Muscle Thickness on Outcomes of Lumbar Fusion Surgery. World Neurosurg..

[32] Urakawa H., Sato K., Vaishnav A.S., Lee R., Chaudhary C., Mok J.K., Virk S., Sheha E., Katsuura Y., Kaito T. (2023). Preoperative cross-sectional area of psoas muscle correlates with short-term functional outcomes after posterior lumbar surgery. Eur. Spine J..

[33] Echt M., Bakare A.A., Varela J.R., Platt A., Abdul Sami M., Molenda J., Kerolus M., Fessler R.G. (2023). Comparison of minimally invasive decompression alone versus minimally invasive short-segment fusion in the setting of adult degenerative lumbar scoliosis: A propensity score-matched analysis. J. Neurosurg. Spine..

[34] Overdevest G.M., Jacobs W., Vleggeert-Lankamp C., Thomé C., Gunzburg R., Peul W. (2015). Effectiveness of posterior decompression techniques compared with conventional laminectomy for lumbar stenosis. Cochrane Database Syst. Rev..

[35] Epstein N.E. (2015). Open laminoforaminotomy: A lost art?. Surg. Neurol. Int..

[36] Holc F., Albani-Forneris A., Kido G., Beltrame S., Petracchi M., Gruenberg M., Sola C., Camino-Willhuber G. (2023). Independent inter and intra-observer agreement of the Schizas’s classification of degenerative lumbar stenosis: Comparison among three levels of surgical training. Rev. Esp. Cir. Ortop. Traumatol..

[37] Kuittinen P., Sipola P., Leinonen V., Saari T., Sinikallio S., Savolainen S., Kröger H., Turunen V., Airaksinen O., Aalto T. (2014). Preoperative MRI findings predict two-year postoperative clinical outcome in lumbar spinal stenosis. PLoS ONE.

[38] Sigmundsson F.G., Kang X.P., Jonsson B., Stromqvist B. (2012). Prognostic factors in lumbar spinal stenosis surgery. Acta Orthop..

